# Retraction: The relationship between spiritual well-being and fear of cancer progression in Iranian cancer patients

**DOI:** 10.3389/fpsyg.2025.1719526

**Published:** 2025-10-13

**Authors:** Frontiers Editorial Office

Following publication, concerns were raised regarding the validity of the data in the article, the ethical approval for this study and the contributions of the authors of the article. An investigation was conducted in accordance with Frontiers' policies.

The authors failed to provide the raw data and a satisfactory explanation during the investigation.

Given the concerns about the study data, authorship, and ethical approval, the data and conclusions of the article have been deemed unreliable and the article has been retracted.

This retraction was approved by the Chief Executive Editor of Frontiers. The authors received a communication regarding the retraction and had a chance to respond. This communication has been recorded by the publisher.

Frontiers would like to thank the concerned reader who contacted us regarding the published article.

